# Understanding the Growth Mechanism of GaN Epitaxial Layers on Mechanically Exfoliated Graphite

**DOI:** 10.1186/s11671-018-2546-x

**Published:** 2018-04-27

**Authors:** Tianbao Li, Chenyang Liu, Zhe Zhang, Bin Yu, Hailiang Dong, Wei Jia, Zhigang Jia, Chunyan Yu, Lin Gan, Bingshe Xu, Haiwei Jiang

**Affiliations:** 1 Key Laboratory of Interface Science and Engineering in Advanced Materials, Taiyuan University of Technology, Ministry of Education, Taiyuan, 030024 China; 2 College of Materials Science and Engineering, Taiyuan University of Technology, Taiyuan, 030024 China; 3 School of Materials Science and Engineering, Huazhong University of Science and Technology, Wuhan, 430074 China; 4 State Key Laboratory of Material Processing and Die and Mould Technology, Huazhong University of Science and Technology, Wuhan, 430074 China; 5 Institute of Atomic and Molecular Science, Shaanxi University of Science and Technology, Xi’an, 710021 China

**Keywords:** Mechanical exfoliation graphite, GaN, Growth mechanism, Highly oriented pyrolytic graphite (HOPG)

## Abstract

The growth mechanism of GaN epitaxial layers on mechanically exfoliated graphite is explained in detail based on classic nucleation theory. The number of defects on the graphite surface can be increased via O-plasma treatment, leading to increased nucleation density on the graphite surface. The addition of elemental Al can effectively improve the nucleation rate, which can promote the formation of dense nucleation layers and the lateral growth of GaN epitaxial layers. The surface morphologies of the nucleation layers, annealed layers and epitaxial layers were characterized by field-emission scanning electron microscopy, where the evolution of the surface morphology coincided with a 3D-to-2D growth mechanism. High-resolution transmission electron microscopy was used to characterize the microstructure of GaN. Fast Fourier transform diffraction patterns showed that cubic phase (zinc-blend structure) GaN grains were obtained using conventional GaN nucleation layers, while the hexagonal phase (wurtzite structure) GaN films were formed using AlGaN nucleation layers. Our work opens new avenues for using highly oriented pyrolytic graphite as a substrate to fabricate transferable optoelectronic devices.

## Background

In the past 20 years, GaN has developed into one of the most important semiconductors after Si because of its excellent optical and electrical properties. As such, GaN has become an attractive material for light-emitting diodes, lasers, and high-power and high-frequency devices [1–5]. Currently, the growth of GaN films by metal-organic chemical vapor deposition (MOCVD) has become the main method of producing large-scale optoelectronic devices [6, 7]. Due to the lack of large native substrates, GaN films are usually grown heteroepitaxially on substrates such as c-sapphire, SiC, or Si. As a result, there is normally a high degree of lattice and thermal mismatching between the GaN films and these substrates, introducing a large number of threading dislocations in the GaN epilayers, which can seriously affect the device’s performance [8–10].

Graphite is a layered structure of hexagonally arranged carbon atoms having strong *σ* bonds within the plane, whereas weak *π* electrons are exposed on the surface [8, 11]. Because the weak van der Waals binding between graphite and GaN films can relax the requirements for lattice matching between two material systems, mechanically exfoliated graphite can be used as an ideal substrate for GaN growth. To date, many studies have reported the successful growth of GaN films on graphene, but the graphene they used is almost always prepared by chemical vapor deposition (CVD) or graphitization of SiC substrates [12–15]. Such graphene layers have abundant step edges and defects that act as nucleation sites to induce film growth.

Highly ordered pyrolytic graphene (HOPG) is a pristine two-dimensional (2D) material, which can be mechanically exfoliated with relative ease to obtain multilayer graphite. This kind of graphite has better crystal quality and photoelectrical properties and can be easily separated from epitaxial films. This is very beneficial to the fabrication of transferable GaN-based devices. However, there are few studies on the growth mechanism by which three-dimensional (3D) films are deposited onto this pristine 2D material. In this paper, the influence of O-plasma treatment and elemental Al addition on the growth of GaN on multilayer graphite is explained based on classic nucleation theory (CNT). This work seeks to promote an understanding of the growth of GaN films on pristine 2D materials.

## Methods/Experimental

### Preparation of Graphite

The graphite was peeled off the HOPG with a Scotch tape; this obtained graphite was first attached to a glass plate coated with a photoresist and heated at 80 °C for 3 min to solidify the photoresist. Then, the graphite left on the photoresist was repeatedly peeled 10 times in the same direction with tape. The last used tape with the thin graphite layer was stuck on a SiO_2_ substrate and then the tape was slowly removed after 10 min. The thin graphite layer left on the SiO_2_ substrate was used for subsequent characterization and GaN growth. This procedure enables control of the graphite thickness within the range of 10 to 20 nm. Finally, the graphite was treated by O-plasma for 40 s at 100 mW.

### Conventional Two-Step Growth (Nucleation at 550 °C and Growth at 1075 °C)

Prior to growth, a cleaning step was performed under H_2_ at 1100 °C for 6 min. This was followed by cooling to the nucleation temperature, and the GaN nucleation layers were grown at 550 °C for 100 s by introducing trimethylgallium (TMGa) and NH_3_ with a flux of 35.7 and 26,800 μmol/min, respectively, at a reactor pressure of 600 mbar. The nucleation layers were annealed at 1090 °C for 2 min, and GaN films were then deposited at 1075 °C for 600 s.

### Modified Two-Step Growth (Nucleation at 1000 °C and Growth at 1075 °C)

The same cleaning step was carried out prior to growth. AlGaN nucleation layers were grown at 1000 °C for 100 s by introducing NH_3,_ trimethylgallium (TMGa) and trimethylaluminum (TMAl) with a flux of 26,800, 22.4, and 13.3 μmol/min, respectively, at a reactor pressure of 100 mbar. The nucleation layers were annealed at 1090 °C for 2 min, and GaN films were then deposited at 1075 °C for 600 s. The growth of AlGaN at low pressures minimizes any pre-reactions between TMAl and NH_3_.

A JSM-6700F field-emission scanning electron microscopy (FE-SEM) from JEOL was used to characterize the surface morphology at each growth stage. A Renishaw Invia Raman spectrometer with a 514-nm excitation laser was used to define the defects in the graphite. Cross-sectional transmission electron microscopy (TEM) images were obtained using focused ion beam milling (FIB; LYRA 3 XMH, TESCAN). Microstructural analysis of GaN films was performed using JEM-2010 high-resolution TEM (HR-TEM). In addition, the SPA-300HV atomic force microscopy (AFM) was used to characterize the roughness of graphite before and after oxygen plasma treatment.

## Results and Discussion

In the general film deposition process, nucleation sites often appear in particular locations on the substrate, such as defects, atomic layer steps, and impurity atoms [16, 17]. These locations can reduce the activation energy for atomic bonding between films and substrate. However, because the pristine graphite surface lacks dangling bonds (indicating chemical inertness), it is difficult for nucleation to occur on the graphite surface.

**Fig. 1:**
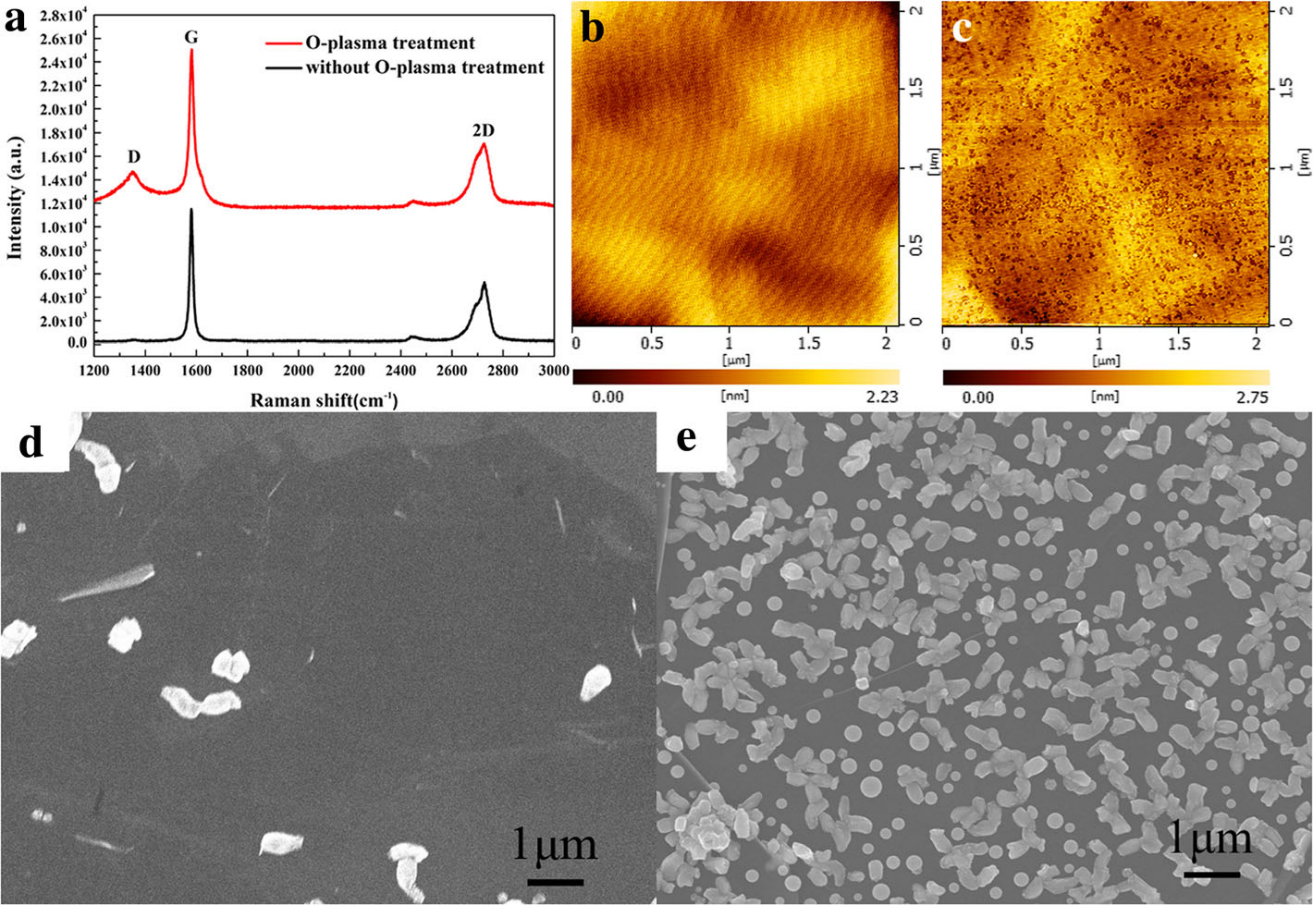
**a** Raman spectra of the untreated graphite (black line) and treated graphite (red line). **b**, **c** 2 × 2 μm^2^ AFM images of the untreated graphite and treated graphite, respectively. **d**, **e** FE-SEM images of nucleation islands grown on untreated graphite and treated graphite, respectively

In order to increase the nucleation density on the 2D graphite surface, O-plasma treatment was used to increase the number of defects by forming the oxygen functional groups on the graphite surface [18], which can promote GaN nucleation on the graphite surface. The typical Raman scattering features of graphite can be observed in Fig. 1a, including the G peak (1582 cm^−1^) and 2D peak (2727 cm^−1^); the intensity ratio between the G peak and 2D peak (*I*_G_/*I*_2D_ = 2.2) denotes the existence of multilayer graphite [19]. The Raman spectra also show an obvious D peak after O-plasma treatment, as shown in Fig. 1a (red line), indicating an increased number of defects compared to the graphite without treatment [20]. As shown in the AFM images in Fig. 1b, c, the roughness of the treated graphite was obviously greater than that of the untreated graphite, as is clear from the root-mean-square (RMS) roughness of the graphite before (0.28 nm) and after (0.39 nm) the treatment; this too reflects the increase in the number of the defects on the graphite surface. Figure 1d, e show SEM images of GaN nuclei islands. Nucleation on the untreated graphite surface was very difficult, and only a few islands of nuclei were formed at the graphite wrinkles, as shown in Fig. 1d. A comparison of Fig. 1d, e shows that the density of the islands increased after O-plasma treatment, which coincides with the Raman spectra and AFM results. The average island size is more than 200 nm in these images, which is larger than the case of nucleation on sapphire using the conventional two-step growth [21]. This is because the low migration barrier of group-III metals on graphite allows atoms to diffuse readily on the surface, which promotes the formation of larger islands [6].

**Fig. 2:**
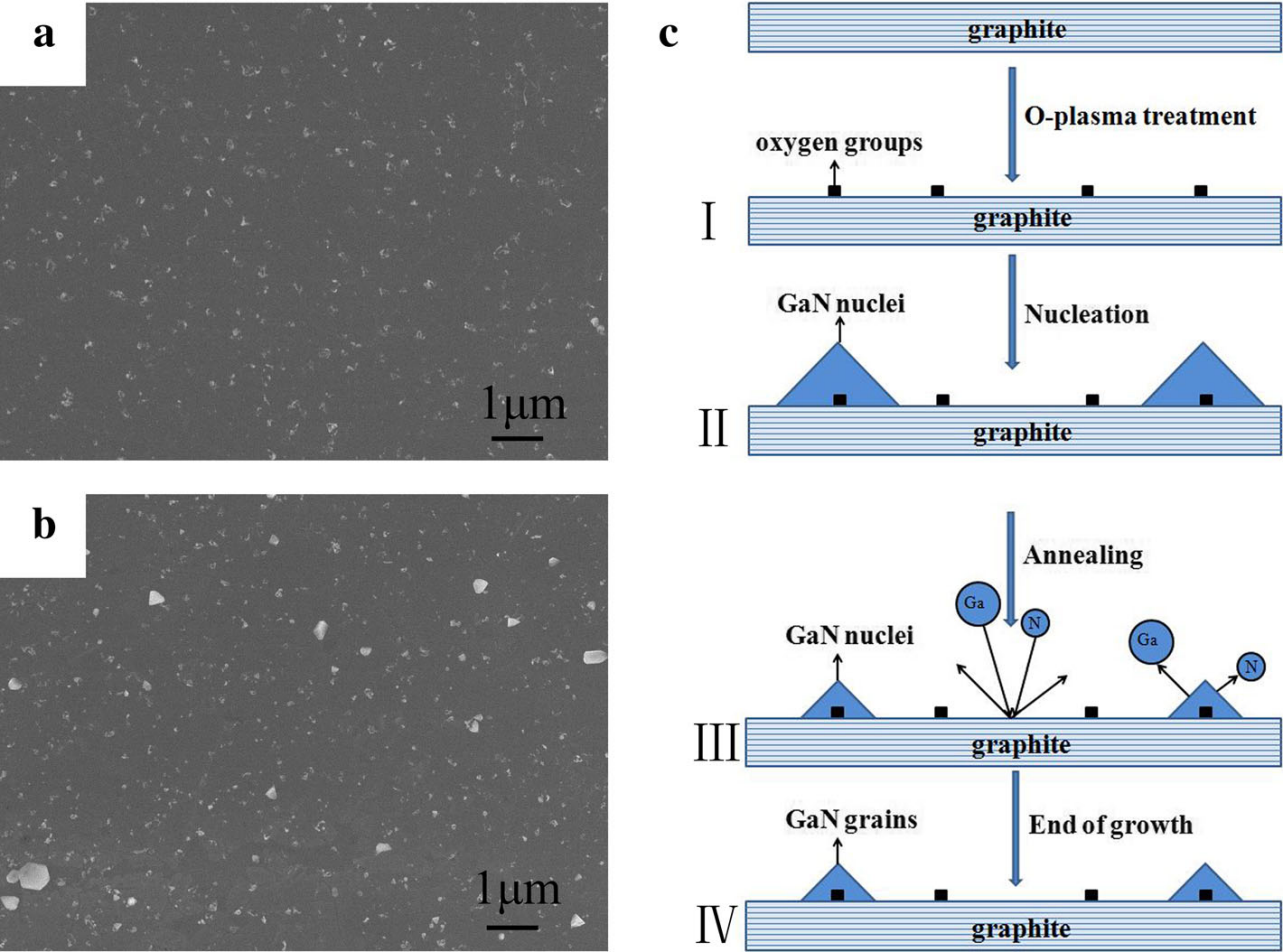
**a** FE-SEM image of annealed islands. **b** The surface morphology of GaN grains at the end of growth. **c** Proposed schematic of growth mechanism of GaN grains

Figure 2a, b show the surface morphology of annealed nuclei islands and GaN grains formed at the end of growth, respectively. As shown in Fig. 2b, only some grains were formed on the graphite surface at the end of the conventional two-step growth. To explore the reason for this phenomenon, interrupted-annealing experiments (i.e., the growth was completely stopped after a certain annealing time) were carried out. A comparison of Fig. 2a with Fig. 1e shows that the density of islands did not change while the island size markedly decreased after annealing.

The GaN growth mechanism on graphite by conventional two-step growth can be explained according to Fig. 2c. The number of defects on the surface of graphite increase after oxygen plasma treatment (Fig. 2c-I). Then, sparse nuclei islands were formed in the subsequent nucleation stage (Fig. 2c-II). These nuclei islands were only decomposed and not re-crystallized during annealing, and their size was significantly reduced, as shown in Fig. 2c-III. We consider that the absence of dense nucleation layers causes nuclei islands to only decompose and fail to re-crystallize in the high-temperature annealing process, which results in a significant reduction in the size after annealing (Fig. 2a). The size of most islands formed at the end of growth did not change significantly after annealing, as shown in Fig. 2c-IV. The reason for this phenomenon is that most of the annealed islands cannot reach the critical radius of Ostwald ripening, and their size did not change during the subsequent growth process [22]. Further, the few islands that reach the critical radius of Ostwald ripening can further adsorb Ga and N atoms, so their size increases with the growth time. However, the density of these islands is too low to form the GaN films, as confirmed in Fig. 2b.

**Fig. 3:**
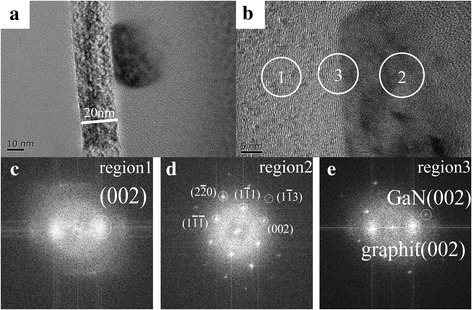
**a**, **b** TEM and HR-TEM cross-section micrographs (along c-GaN [110]) showing the GaN and graphite interface. **c**–**e** Fourier transform diffraction patterns for graphite, GaN grains, and their interface, respectively

The GaN grains obtained by conventional two-step growth were characterized by HR-TEM to investigate their microstructures. Figure 3a clearly indicates the presence of multilayer graphite, where the graphite thickness is 20 nm and the size of GaN grains grown on the graphite surface is approximately 20 nm. Figure 3b shows an HR-TEM cross-section micrograph of the GaN-graphite interface. Fast Fourier transform (FFT) diffraction patterns for the graphite (region 1) clearly show the (002) plane of graphite (Fig. 3c). The entire GaN grain presents only the cubic structure, as per the FFT diffraction patterns of region 2 (Fig. 3d), which confirms that our nuclei islands only undergo decomposition and not re-crystallization during the annealing process, as shown in Fig. 2c. This result is inconsistent with that reported in reference [23]. In their study, the cubic GaN containing with hexagonal phase at their top was formed owing to the occurrence of decomposition and re-crystallization during the annealing process. As shown in Fig. 3e, this cubic GaN grain did not grow along with the graphite (002) plane.

**Fig. 4:**
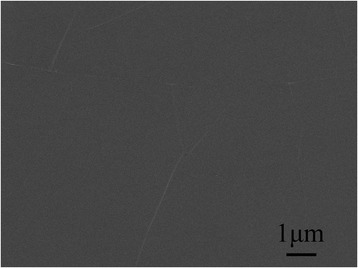
FE-SEM images of nucleation layers grown at 1000 °C

As demonstrated above, GaN films cannot be deposited on graphite surfaces by conventional two-step growth. We therefore attempted to solve this problem by increasing the nucleation temperature at a fixed nucleation density, owing to the improved ability of atoms to migrate at high temperatures. Experiments were therefore carried out at a high nucleation temperature of 1000 °C, which indicated that GaN could not be formed on the graphite surface even at this high temperature, as shown in Fig. 4. Generally, high temperatures have a trade-off effect on the nucleation process based on the CNT [24]. While high temperatures can promote the migration of atoms, it is known that the nucleation rate (*dN*/*dt*) decreases at high temperatures according to the nucleation rate formula:

$$ \frac{dN}{dt}\propto \exp \left[\frac{\left({E}_{\mathrm{d}}-{E}_{\mathrm{s}}-\Delta  {G}^{\ast}\right)}{kT}\right] $$

where *N* is the number density of growing centers [25], *E*_d_ is adsorption energy, *E*_S_ is activation energy for migration, ΔG* is the nucleation barrier, *T* is the absolute temperature, and *k* is the Boltzmann constant. Moreover, high-temperature conditions reduce the sticking coefficient of graphite. We consider that the low nucleation rate and sticking coefficient at high temperatures play a determinate role in the nucleation stage, preventing the GaN nuclei from forming on the graphite surface.

Based on the nucleation rate formula, we sought to improve the nucleation rate by increasing the adsorption energy and reducing migration barrier between nucleation layers and graphite at high temperatures. In addition, Al has a higher adsorption energy (1.7 eV) and a lower migration barrier (0.03 eV) on the graphite surface than Ga (the adsorption energy and the migration barrier of Ga atoms is 1.5 and 0.05 eV, respectively) based on previous studies [26]; Al hardly desorbs from graphite surfaces and easily migrates on it, which can increase the nucleation rate. AlGaN nucleation layers were thus adopted in subsequent experiments.

**Fig. 5:**
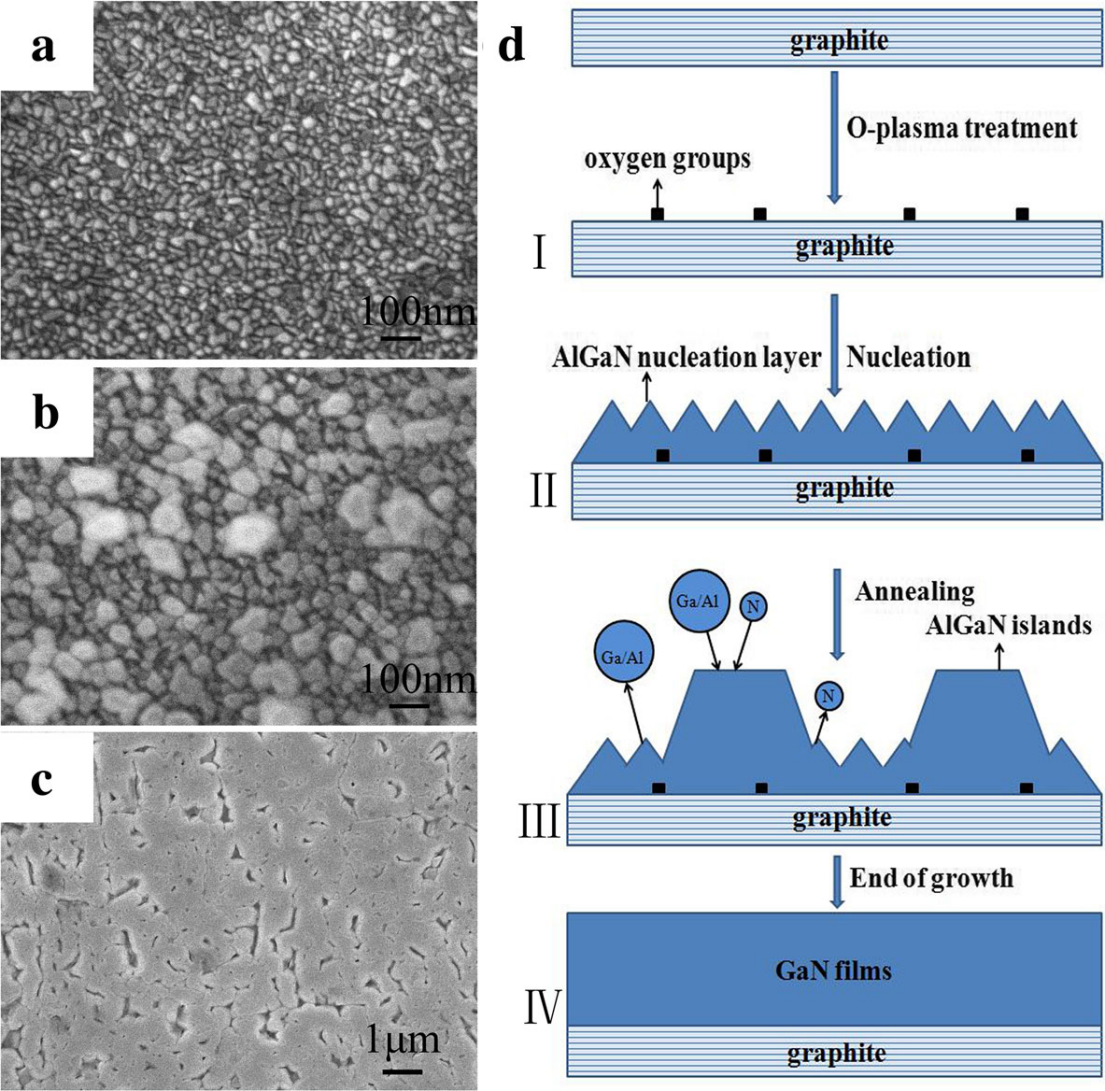
**a**–**c** FE-SEM images of nucleation layers, annealed layers, and epitaxial layers, respectively. **d** Schematic of the corresponding growth mechanism of GaN films using AlGaN nucleation layers

The formation of GaN films using AlGaN as the nucleation layers by modified two-step growth coincided with the growth mechanism shown in Fig. 5d. The nucleation rate increased with the addition of Al to the nucleation layers, resulting in the formation of dense nucleation layers at the same nucleation density (Fig. 5d-II), which was confirmed by the SEM image of the nucleation layers (Fig. 5a). The dense nucleation layers provide abundant adsorption sites, which is beneficial for the re-crystallization of Ga and N atoms to form large nuclei islands, as shown in Fig. 5d-III. Hence, the islands (3D) became larger after high-temperature annealing (Fig. 5b). Based on the formation of large islands, the coalescence of these islands easily occurs during subsequent growth, which leads to the quasi-two-dimensional growth of GaN films (2D) as depicted in Fig. 5c.

**Fig. 6:**
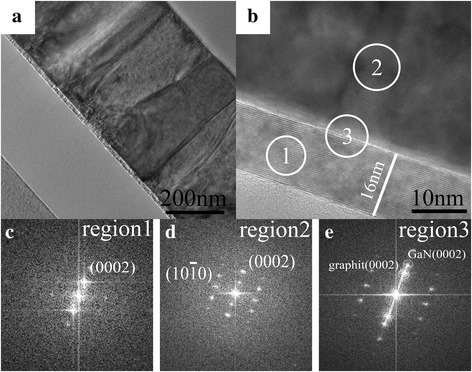
**a**, **b** TEM and HR-TEM cross-section micrographs (along h-GaN [010]) showing the GaN and graphite interface. **c**–**e** Fourier transform diffraction patterns for graphite, GaN films, and their interface, respectively

The microstructure of the GaN films was further investigated by TEM. The GaN graphite heterostructure is clearly visible in Fig. 6a, b. Figure 6a shows that the graphite layer’s thickness is 16 nm, and it also shows the grain boundary formed by the coalescence of nuclei islands, where the diameter of each grain coincides with the size of the nuclei island shown in Fig. 5b. FFT diffraction patterns for the graphite layer (region 1) clearly show the (0002) plane of graphite (Fig. 6c), while FFT diffraction patterns for GaN films (region 2) exhibit regular spot arrays of hexagonal (wurtzite) GaN (Fig. 6d). Further, the FFT diffraction patterns of the interface (region 3) indicate that GaN films grew along the graphite (0002) plane (Fig. 6e). It has been reported that the AlN nucleation layers is predominantly the wurtzite (hexagonal) phase [23]. Based on our experiment results, it can be concluded that the nucleation layers tend to form the hexagonal structure when Al is added to it, which allows the subsequent growth of the GaN films with the hexagonal structure.

## Conclusions

The effects of O-plasma treatment and elemental Al addition on the growth of GaN films on pristine graphite were studied based on CNT. The introduction of defects by O-plasma treatment reduces the activation energy needed for atomic bonding, increasing the nucleation density of the graphite surface. In addition, adding Al can effectively improve the nucleation rate due to its high adsorption energy and low migration barrier with graphite, thus forming dense nucleation layers and promoting the subsequent growth of GaN films. This study accelerates the fabrication of optoelectronic devices using high purity graphite as a substrate.

## Abbreviations

2D: two-dimensional
3D: three-dimensional
AFM: Atomic force microscopy
CNT: Classic nucleation theory
CVD: Chemical vapor deposition
FE-SEM: Field-emission scanning electron microscopy
FFT: Fast Fourier transform
HOPG: Highly oriented pyrolytic graphite
HR-TEM: High-resolution transmission electron microscopy
MOCVD: Metal-organic chemical vapor deposition
